# Rapid genome-scale mapping of chromatin accessibility in tissue

**DOI:** 10.1186/1756-8935-5-10

**Published:** 2012-06-26

**Authors:** Lars Grøntved, Russell Bandle, Sam John, Songjoon Baek, Hye-Jung Chung, Ying Liu, Greti Aguilera, Carl Oberholtzer, Gordon L Hager, David Levens

**Affiliations:** 1 Laboratory of Receptor Biology and Gene Expression, 41 Library Dr. National Cancer Institute, Building 41 B602, Bethesda, MD, 20892, USA; 2 Gene Regulation Section Laboratory of Pathology, National Cancer Institute, NIH, Building 10, Room 2 N106, Bethesda, MD, 20892, USA; 3 Section on Endocrine Physiology, National Institute of Child Health and Development, NIH, Building 10, Room 1-3330, Bethesda, MD, 20892, USA; 4 Laboratory of Pathology, National Cancer Institute, NIH, Building 10, Room 2 N212, Bethesda, MD, 20892, USA

**Keywords:** Chromatin accessibility, Tissue, TACh, Benzonase, Cyanase, DNase I

## Abstract

**Background:**

The challenge in extracting genome-wide chromatin features from limiting clinical samples poses a significant hurdle in identification of regulatory marks that impact the physiological or pathological state. Current methods that identify nuclease accessible chromatin are reliant on large amounts of purified nuclei as starting material. This complicates analysis of trace clinical tissue samples that are often stored frozen. We have developed an alternative nuclease based procedure to bypass nuclear preparation to interrogate nuclease accessible regions in frozen tissue samples.

**Results:**

Here we introduce a novel technique that specifically identifies Tissue Accessible Chromatin (TACh). The TACh method uses pulverized frozen tissue as starting material and employs one of the two robust endonucleases, Benzonase or Cyansase, which are fully active under a range of stringent conditions such as high levels of detergent and DTT. As a proof of principle we applied TACh to frozen mouse liver tissue. Combined with massive parallel sequencing TACh identifies accessible regions that are associated with euchromatic features and accessibility at transcriptional start sites correlates positively with levels of gene transcription. Accessible chromatin identified by TACh overlaps to a large extend with accessible chromatin identified by DNase I using nuclei purified from freshly isolated liver tissue as starting material. The similarities are most pronounced at highly accessible regions, whereas identification of less accessible regions tends to be more divergence between nucleases. Interestingly, we show that some of the differences between DNase I and Benzonase relate to their intrinsic sequence biases and accordingly accessibility of CpG islands is probed more efficiently using TACh.

**Conclusion:**

The TACh methodology identifies accessible chromatin derived from frozen tissue samples. We propose that this simple, robust approach can be applied across a broad range of clinically relevant samples to allow demarcation of regulatory elements of considerable prognostic significance.

## Background

The coupling of next-generation sequencing methodologies with classical enzymatic chromatin digestion approaches (eg. DNase I and MNase) has provided global, high-resolution information on chromatin features such as nucleosome positioning and chromatin accessibility [1,2]. DNase-Seq has emerged as a powerful genome-wide tool to identify and characterize chromatin transitions (DNase I hypersensitive sites or DHS) in regulatory regions across a range of biological processes and cell lines [1,3-5]. Current methodologies to generate genome-wide DHS data start with large numbers of fresh cells from which sizeable quantities of high quality nuclei need to be generated [6]. This has restricted application of the DNase I approach to cells in culture and freshly isolated tissue and has prevented the use of this technology on clinical tissue samples that are typically scarce, stored frozen and from which nuclei are difficult to obtain.

The use of DNase I as a probe of chromatin accessibility was initially described three decades ago [7,8], where partial digestion of chromatin with DNase I indentified DHS in promoters of heat shock genes. The nucleosomal steric hindrance of DNase I access to DNA is similar to that of transcription factors (TF) and the degree of DNase I accessibility correlates with the level of TF occupancy [9]. Thus DNase I hypersensitivity is considered an independent, unbiased probe for TF accessibility. Today, the primary commercial sources of DNase I are either recombinant proteins or purified enzymes from bovine pancreas. However, DNase I is known to have drawbacks that restrict its broad-spectrum use. Specifically, it is known to have a narrow effective concentration window, requiring fastidious titration of enzyme and chromatin to obtain optimal partial digestion [6,7]. Moreover, DNase I is inhibited by high levels of actin [10] and because the actin content varies amongst cell and tissue types, each biological sample must be individually titrated. Preparation of nuclei prior to digestion reduces actin contamination, while *in situ* NP40 lysis leaving adherent nuclei also allows DNase I treatment of cells grown as a monolayer [11].

In order to define accessible chromatin compartments in samples derived from frozen tissue, where cell numbers are unknown and nuclear preparation is problematic, we hypothesized that digestion of chromatin with more robust nucleases that function in a coarse environment as well as, over broad concentration ranges, might enable the determination of chromatin accessibility in tissue samples. Benzonase, a recombinant endonuclease derived from *Serratia marcescens*, is composed of two identical subunits of 30 kDa, requires divalent cations for full activity and is generally used to clear cellular protein extracts of DNA and RNA prior to downstream analysis [12]. It digests DNA and RNA efficiently under a range of conditions to nucleotides of 2–5 base pairs. Although the enzyme is able to cleave DNA at all positions, it has been reported to have a relative preference for GC rich regions over dA/dT tracts [13]. Cyanase is a less described non-Serratia recombinant endonuclease that, like Benzonase, efficiently digests DNA and RNA under harsh conditions. Here we introduce a novel technique that combines rapid processing of frozen tissue using Benzonase and Cyanase to specifically identify Tissue Accessible Chromatin (TACh) from frozen specimens. Accessible regions identified with TACh correlate with features of euchromatin and levels of transcription, suggesting that these accessible regions are indeed regulatory. We propose that TACh will be a valuable tool to identify the physiological or pathological regulatory features of chromatin from clinical materials.

## Results

### Benzonase and Cyanase as probes for chromatin accessibility

**Figure 1:**
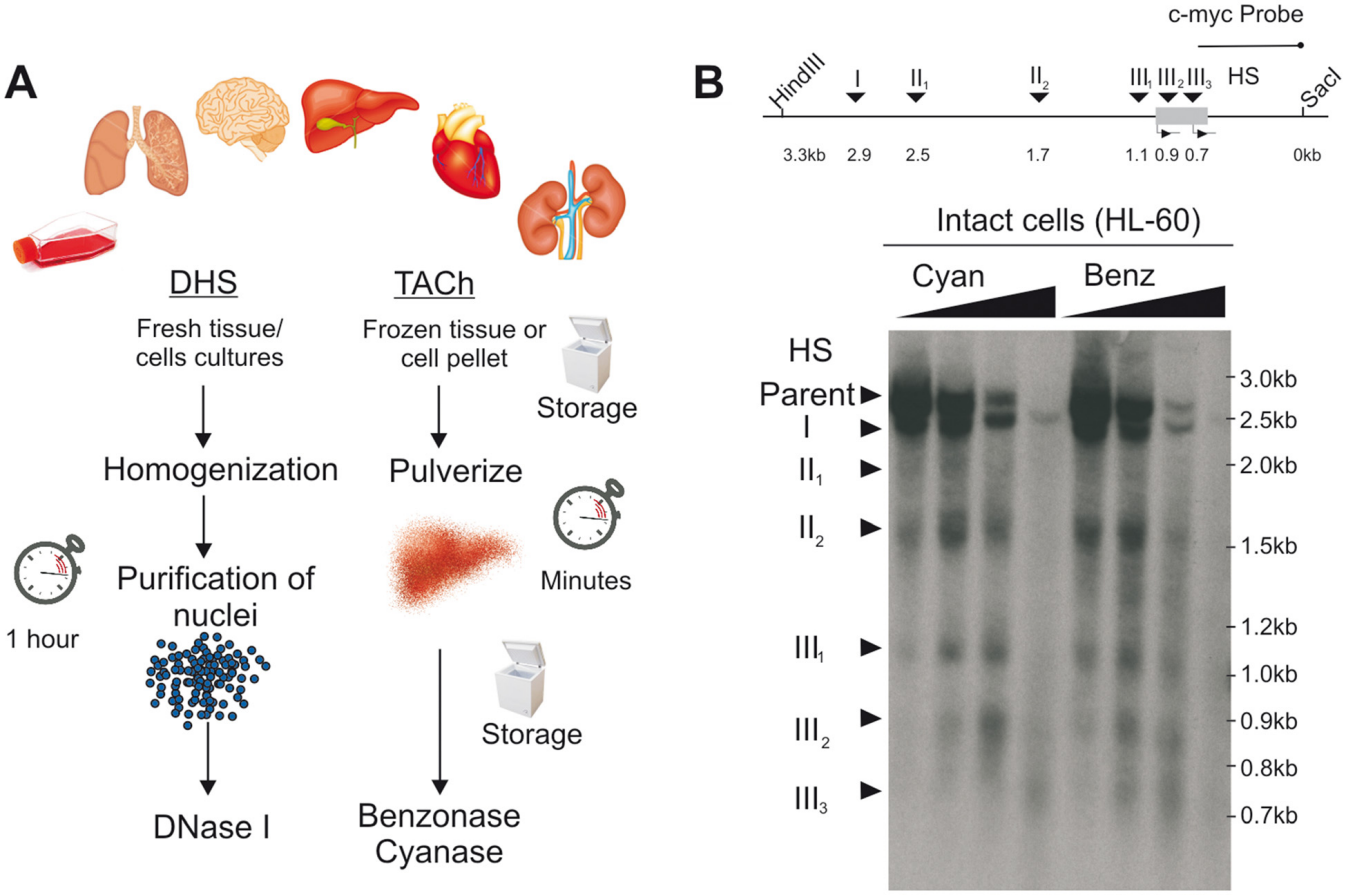
**Identification of nuclease hypersensitive regions in chromatin using TACh.(A)** Side by side comparison of experimental setups for identification of nuclease hypersensitive sites using DNase I (DHS) versus Benzonase-Cyanase (TACh). (left panel) For DHS freshly isolated tissue is homogenized and nuclei are purified using protocols that take one to two hours. Purified nuclei are treated with various concentrations of DNase I. DNA fragments released by partial endonucleae digestion are isolated and can be analyzed by Southern blotting, qPCR or deep sequencing. (right panel) In contrast to the DHS procedure, TACh uses frozen tissue or cell pellets as starting material. Tissue is initially pulverized and divided into appropriate quantities, which can be stored frozen. Pulverized tissue is suspended in hypotonic nuclease buffer containing Benzonase or Cyanase at various concentrations and like for the DHS assay, DNA fragments released by partial endonuclease digestion can be analyzed by the same various methods. **(B)** Identification of nuclease accessible regions at the c-myc promoter in intact human HL60 cells using Benzonase or Cyanase. Detection of accessibility by Southern blotting and indirect end labeling is shown. (Top) Illustration of the human c-myc promoter with indication of probe annealing site and six previously published DNase I hypersensitive (HS) sites. (Bottom) Intact HL-60 cells, grown in suspension, were resuspended in hypertonic nuclease digestion buffer containing Benzonase or Cyanase. Digested DNA was purified and digested with HindIII and SacI, which releases a 3.3 kb fragment of the c-myc promoter. Partial nuclease digested fragments were detected using a biotinylated probe as indicated.

Accessible chromatin has traditionally been identified by DNase I digestion of chromatin using nuclei as starting material (Figure 1A, left panel). Although nuclei can be very efficiently purified from cell lines and fresh tissue within one to two hours, such purification requires disassociation of cells, and washing by centrifugation, conditions that could modify signaling to the nucleus or allow leaching of chromatin-bound components, potentially altering nuclear structures. Extracting nuclei from frozen tissue samples is even more cumbersome and complicated. Thus, in order to minimize the time between the snap freezing of tissue and enzymatic digestion, we have developed a method that avoids nuclear preparation and uses a different endonucleaese, Benzonase or Cyanase, to digest accessible chromatin-embedded DNA (Figure 1A, right panel).

To set a standard for the fidelity of Benzonase and Cyanase as a probe for chromatin accessibility, we initially performed a conventional nuclease hypersensitivity assay using cultured cells. Human promyelocytic leukemia cells (HL-60) grown in suspension were isolated, resuspended hypotonic buffer and incubated with increasing concentrations of Benzonase and Cyanase. Accessible regions at the c-myc promoter were compared using indirect-end labeling and Southern blotting as previously described [14]. We show that Benzonase and Cyanase yielded the same pattern of hypersensitive regions expected for DNase I [14], demonstrating that Benzonase and Cyanase are useful probes for chromatin accessibility (Figure 1B).

### Identification of accessible chromatin in frozen tissue

**Figure 2:**
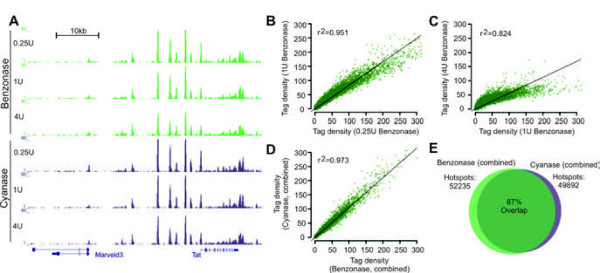
**TACh on mouse liver tissue with three different concentrations of Benzonase and Cyanase.** Partially digested DNA was Illumina sequenced and aligned to the mouse mm9 genome assembly. **(A)** Example of aligned sequence tags at a region surrounding the Tat gene. **(B-C)** Scatter plot comparing tag density of Benzonase hotspots at different enzyme concentrations. Linear correlation coefficients are indicated. **(D)** Scatter plot comparing tag density of identified hotspots when all three Benzonase and Cyanase concentrations were combined. The linear correlation coefficient is indicated. **(E)** When tags from all three Benzonase and Cyanase concentrations were combined, Benzonase identified 52235 hotspots and Cyanase 49892 hotspots. 87% of these hotspots were identified by both enzymes.

To test whether Benzonase and Cyanase can interrogate nuclease accessible regions in chromatin from frozen tissue, whole livers from C57BL/6 mice were isolated and frozen immediately in liquid nitrogen. We initially compared different procedures to prepare frozen tissues amenable for nuclease treatment without disrupting chromatin integrity. We found that rapid pulverization of frozen tissue into a fine powder prior to digestion results in the best signal to noise ratio (data not shown). Pulverization was performed on dry ice with equipment pre-cooled in liquid nitrogen and pulverized tissue was stored as aliquots (Figure 1A, right panel). For digestion, pulverized tissue was directly resuspended in a hypotonic digestion buffer and subsequently incubated with Benzonase or Cyanase at different concentrations. DNA fragments from chromatin digested with 0.25U/ml, 1U/ml and 4U/ml of Benzonase or Cyanase were isolated as previously described [1], sequenced to a depth of 20–30 million uniquely aligning tags (Additional file 1: Figure S 1) and accessible regions were identified as previously described [1]. At all three enzyme concentrations, Benzonase and Cyanase revealed a robust set of nuclease hypersensitive regions in the genome as exemplified by the tyrosine aminotransferase (Tat) gene, a highly expressed liver-specific gene (Figure 2A). Reflecting the usage of frozen tissue, a larger portion of tags generated from TACh aligns with the mitochondrial genome compared to tags generated by DNase I digestion of chromatin from nuclei (Additional file 1: Figure S 1). Genome-wide, the tag density of hotspots identified with 0.25U of Benzonase resembled the tag density of hotspots identified with 1U of Benzonase, correlation coefficient of 0.951 (Figure 2B), suggesting that a fourfold increase in enzyme concentration identifies the same spectrum of hotspots. When the enzyme concentration was increased an additional fourfold to 4U/ml, although the most intense hotspots were reduced in intensity the overall correlation was still 82% with 1U/ml enzyme (Figure 2C). Similar patterns were seen using Cyanase (Additional file 1: Figure S 2) and remarkably at the different enzyme concentrations both enzymes performed very similarly (Additional file 1: Figure S 3). When data was combined from all three concentrations of Benzonase and Cyanase, each identified ~50,000 hotspots with remarkably similar tag densities (Figure 2D, correlation coefficient of 0.973) and an 87% overlap (Figure 2E). Thus in contrast to the narrow concentration windows of DNase I needed for optimal digestion [14], the hotspot patterns obtained with Benzonase-Cyanase were robustly conserved over a 16-fold range in enzyme concentration. This is a notable advantage for the use of Benzonase or Cyanase with frozen tissue or cell samples when exact cell counts are unavailable.

### Correlation of Benzonase hotspots with euchromatin and TSS of active genes

**Figure 3:**
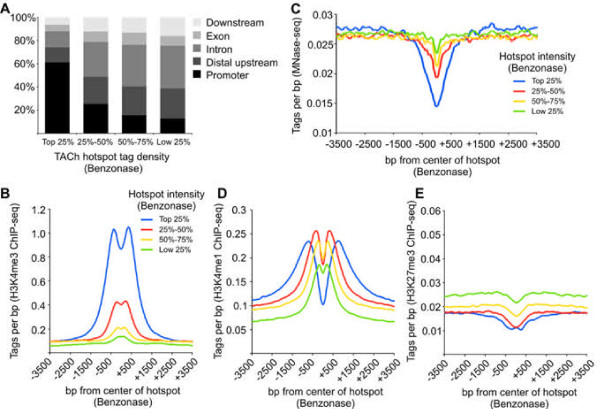
**Correlation of Benzonase accessible regions with epigenetic marks.** Tags from all three Benzonase concentrations were combined and identified hotspots were divided into quartiles according to the hotspots tag density, where the top 25% represents the most intense hotspots and the low 25% represents the least intense hotspots. **(A)** Distribution of hotspots in promoters (2 kb upstream TSS), distal/intergenic regions, introns, exons and downstream regions (2 kb downstream TTS). Tag density of H3K4me3 **(B)**, H3K4me1 (**D**), H3K27me3 (**E**) 3.5 kb up and downstream from Benzonase hotspots. **(C)** Liver MNase-seq tag density surrounding the Benzonase hotspots.

To verify that the TACh procedure identifies accessible regions associated with regulatory elements of gene expression, we mapped the distribution of hotspots in distal upstream regions, promoters, introns, exons and downstream regions, and correlated hotspot intensity with previously mapped histone modifications [15] and nucleosome occupancy [16] in mouse liver tissue (Figure 3). The hotspots with the highest tag densities were found primarily at promoters, whereas the weaker hotspots located mainly in distal upstream and intronic regions similar to enhancers and other regulatory elements (Figure 3A). In agreement, the most intense hotspots were flanked by the promoter-specific H3K4me3 histone modification compared to less intense hotspots (Figure 3B). Moreover the most intense hotspots were also the most sensitive to MNase digestion, suggesting that these regions are either nucleosome –free or occupied by highly mobile nucleosomes flanked by H3K4me3-modified nucleosomes (Figure 3C). H3K4me1, present at promoters as well as enhancers, was enriched at both strong and weak Benzonase hotspots (Figure 3D), while H3K27me3, associated with heterochromatic regions, was deficient at Benzonase hotspots (Figure 3E). Thus Benzonase accessibility is associated with euchromatic features, demonstrating that the TACh method identifies accessible regulatory regions of the genome from frozen tissue.

**Figure 4:**
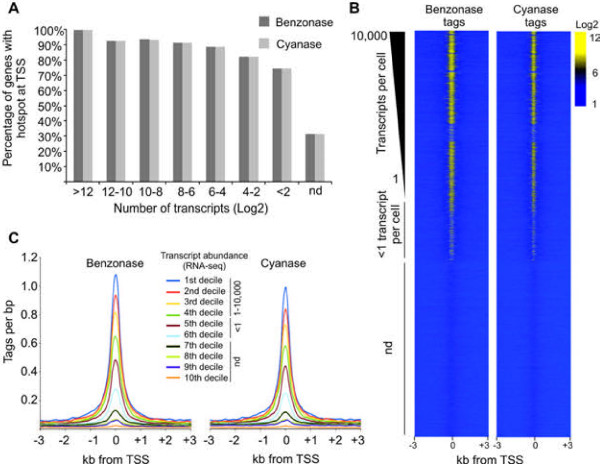
**Benzonase accessibility of TSS. The Level of accessibility correlates with levels of gene transcription.(A)** Transcripts from annotated genes were divided into eight bins according to mRNA abundance (transcripts pr cell, Log2) based on mouse liver RNA-seq data [18]. Genes producing no detectable transcripts are labeled nd (not detected). All TSSs from individual genes were extracted from the UCSC genome browser and related to Benzonase and Cyanase hotspots. Genes with multiple TSS were scored when at least one of the TSSs overlapped with a nuclease hotspot. **(B)** Genes were ranked according to transcript abundance and tag densities from Benzonase and Cyanase digestions (all concentrations combined) were measured 3 kb up and downstream of the corresponding TSSs and visualized as heatmaps. **(C)** Transcripts from annotated genes were divided into deciles according to mRNA abundance (transcripts pr cell), where the first to fourth decile represent genes with more than one transcript pr cell. Deciles five and six contain genes with less than one transcript pr cell and decitile seven to ten contains genes that are not transcribed. Tags sequenced from Benzonase and Cyanase digestions (all concentrations combined) were measured 3 kb up and downstream of the corresponding TSSs and visualized as compiled histograms.

Transcriptional start sites (TSS) of active genes are occupied by highly mobile nucleosomes [17] and are thus highly accessible to DNase I. In agreement, more than 90% of genes producing more than 16 transcripts were marked by Benzonase and Cyanase hotspots at the TSS (Figure 4A); conversely, only 30% of TSSs of inactive genes contained Benzonase-Cyanase hotspots (Figure 4A). Moreover, active genes had an overall increase in Benzonase and Cyanase accessibility at TSSs, compared to less active or silent genes (Figure 4B). Furthermore, when TSSs were binned into deciles according to the abundance of their gene transcripts, measured by previously published RNA-seq data [18], a positive correlation of gene transcription with the degree of Benzonase and Cyanase accessibility was observed (Figure 4C).

### Benzonase and Cyanase accessible regions overlap with DNase I hotspots

**Figure 5:**
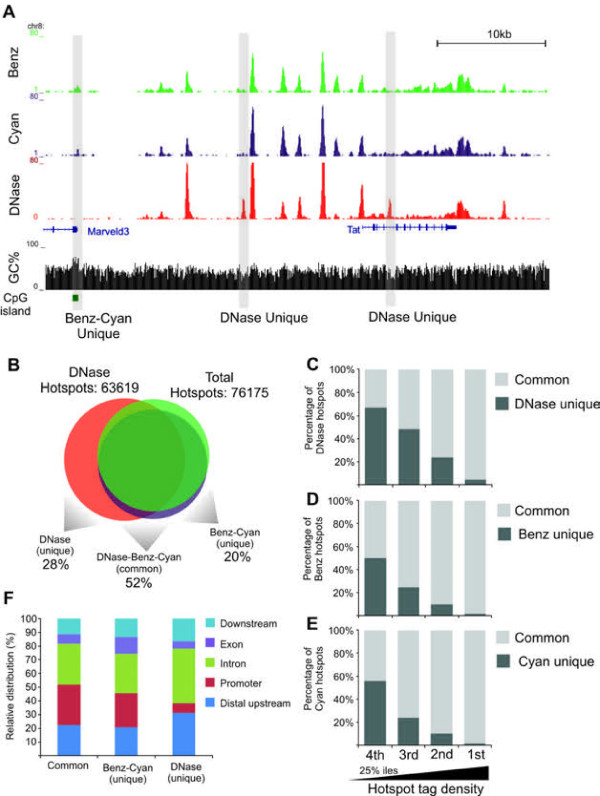
**Direct comparison of hotspots identified by DNase I, Benzonase and Cyanase in the mouse liver genome.(A)** Example of aligned sequence tags at a region surrounding the Tat gene. Aligned tags from Benzonase and Cyanase and DNase I are illustrated together with presence of GpC islands (green boxes) and GC percentage in a 10 bp widow. Benzonase-Cyanase and DNase I unique hotspots are indicated. **(B)** Venn diagram illustrating overlap between regions accessible to DNase I, Benzonase and Cyanase. A total of 63619 hotspots were identified from chromatin digested with DNase I (tags combined from two replicates). Combined with the Benzonase and Cyanase data a total of 76176 nuclease hotspots were identified. 28% of these are unique to DNase I, 52% are identified by DNase I and Benzonase-Cyanase and 20% are unique to Benzonase-Cyanase. (**C-E**) Hotspots identified by DNase I (**C)**, Benzonase **(D)** and Cyanase **(E)** were divided into quartiles according to tag density of hotspots, where the first 25%-ile is the most intense hotspots and the fourth 25%-ile is the least intense hotspots. Hotspots identified by DNase, Benzonase and Cyanase (common) and nuclease unique hotspots were quantified and visualized as percentage of total hotspots identified by DNase I (**C**), Benzonase (**D**) and Cyanase (**E**).

To validate that accessible regions identified by the TACh are indeed bona fide nuclease hypersensitive sites, we mapped DNase I accessible regions using nuclei purified from fresh liver tissue (Figure 1A, left panel). Benzonase, Cyanase and DNase I accessible regions were largely similar at the Tat gene locus. However, we also observed features unique to each nuclease (Figure 5A). Using identical parameters to identify hotspots we detected ~63,000 DNase I hotspots (Figure 5B) which combined with the Benzonase and Cyanase data, identified a total of ~76,000 hotspots. Of these 28% was unique to DNase I, 52% was shared among the three enzymes and 20% was unique to Benzonase-Cyanase (Figure 5B). Parsing nuclease hotspots into quartiles according to tag density, we observed that 62% of the weakest DNase I hotspots were unique whereas 97% of the strongest hotspots overlapped with Benzonase-Cyanase hotpots (Figure 5C). Likewise ~50% of the least intense Benzonase and Cyanase hotspots were unique while close to all of the most intense hotspots overlapped with DNase I hotspots (Figure 5D-E). This suggests that most of highly accessible regions are identified by all enzymes whereas less accessible regions may be unique to particular nucleases. Alternatively many of these less accessible unique regions may have their origin in background digestion by the nucleases and may not be significant. Furthermore Dnase I unique hotspots were preferentially found at introns and distal regions in contrast to Benzonase-Cyanase hotspots which were enriched at promoters (Figure 5F).

### Sequence bias for endonucleases

**Figure 6:**
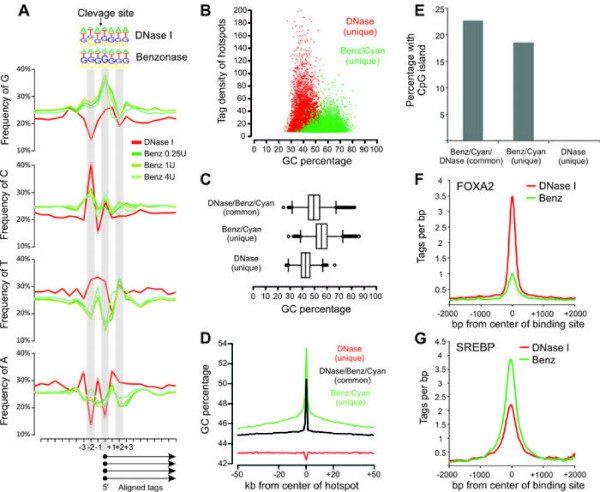
**Biochemical difference between Benzonase, Cyanase and DNase I correlates with TACh preference for GC rich regions.(A)** Frequency of bases at the 5’ end of all aligned tags from chromatin digestion with DNase I (red) and Benzonase (0.25U/ml, 1U/ml, 4U/ml, greens). **(B)** Scatter plot illustrating tag density of DNase I and Benzonase-Cyanase unique hotspots relative to GC content of hotspots. **(C)** Box plot illustrating GC content of DNase I/Benzonase/Cyanase (common), DNase I unique and Benzonase/Cyanase unique hotspots. **(D)** GC content surrounding DNase I/Benzonase/Cyanase (common), DNase I unique and Benzonase/Cyanase unique hotspots. **(E)** Percentage of DNase I/Benzonase/Cyanase (common), DNase I unique and Benzonase/Cyanase unique hotspots harboring a CpG island. **(F)** Compiled sequenced tags from DNase I and Benzonase chromatin digestions, 2 kb up and downstream of previously reported FOXA2 and SREBP-2 binding sites in liver.

The variation observed between identified hotspots by the nucleases might be explained by the intrinsic methodological differences between TACh and the DNase I-based assays. Specifically, TACh is performed in intact cells with minimum manipulation prior to digestion, while the DNase I assay is performed on nuclei that take at least an hour to process. Alternatively, differences between DNase I, Benzonase and Cyanase can be a consequence of sequence specificity for DNA recognition and cleavage by each of the endonucleases. Benzonase preferentially digests dsDNA enriched for Gs and Cs [13] while DNase I prefers Ts [19]. In agreement with the base-specificity explanation, Benzonase and Cyanase unique hotspots at the Tat loci overlapped with a GC-rich CpG island proximal to the Marveld3 gene, whereas DNase I unique hotspots overlapped with low-GC regions (Figure 5A). To explore sequence selectivity for cleavage genome-wide, we analyzed the sequence immediately upstream and downstream of all tags sequenced after digestion with DNase I or Benzonase. As shown in Figure 6A, the sequence tags yielded by Benzonase digestion were enriched for Gs at their 5’ ends, whereas the tags produced by DNase I digestion were enriched for 5’ Ts, suggesting that Benzonase-Cyanase preferentially cleaved at accessible DNA regions with high GC content and DNase I at accessible regions with high AT content. In agreement, the hotspots unique to Benzonase-Cyanase had higher overall GC content compared to surrounding regions (Figure 6D) or DNase I unique hotspots (Figure 6B-C). In contrast, DNase I unique hotspots had higher AT content than either neighboring regions or Benzonase-Cyanase hotspots (Additional file 1: Figure S 4). Common hotspots identified by all three enzymes had intermediate GC contents (Figure 6C). Consistent with the preference of Benzonase-Cyanase for high GC content regions, about 23% of hotspots uniquely identified by Benzonase and Cyanase were within CpG islands, whereas less than 1% of hotspots unique to DNase I mapped to CpG islands (Figure 6E). Benzonase-Cyanase specific hotspots were found to a greater extent within promoters (Figure 5F) and the average GC content of these unique Benzonase-Cyanase accessible promoters was 61% with 57% of them overlapping with CpG islands.

Nuclease accessible sites are maintained by the targeted recruitment of remodeling complexes by transcription factors [20]. The transcription factor FOXA2 has been shown to bind AT rich DNA motifs [21], whereas SREBP has been suggested to bind regions together with SP1 [22] with a resulting preference for GC-rich motifs embedded in CpG islands. Accordingly, the tags sequenced following DNase I digestion were recovered at higher rates from FOXA2 binding sites compared to the tags sequenced using Benzonase-Cyanase digestion (Figure 6F). In contrast, tags from Benzonase-Cyanase digestion were recovered more efficiently at SREBP binding sites (Figure 6G), emphasizing that the differences in base-selectivity at cleavage sites for these enzymes translates into differentially efficient capacities to interrogate the compartments associated with different transcription factors. Though largely similar, Benzonase-Cyanase and DNase I possess unique features that widen the capacity for genome-wide interrogation of chromatin accessibility.

## Discussion

Identification of nuclease accessible sites is a powerful approach to annotate regulatory regions of the genome. In cell lines and fresh tissue where nuclei can be isolated with high efficiency, DNase I has been used for decades to probe for accessibility. However DNase I is inhibited by actin and DNase I can therefore not be used on frozen tissue samples where nuclei cannot be purified in sufficient amounts while maintaining nuclear integrity. Here, we show the use of two other nucleases, Benzonase and Cyanase, in probing chromatin accessibility in frozen whole tissue samples. The digestion patterns of chromatin with Benzonase and Cyanase are remarkably similar and the identified accessible regions correlate with low nucleosomal occupancy, epigenetic marks of euchromatin and levels of transcription of proximal genes, validating the use of TACh for identification of regulatory elements in the genome. Comparisons with DNase I accessibility in liver tissue display a significant overlap between DNase I accessible sites and accessible regions identified by TACh. The similarities are most pronounced at highly accessible regions, whereas identification of less accessible regions tends to be more divergent between nucleases. Interestingly, we show that some of the differences between DNase I and Benzonase relate to their intrinsic sequence biases, with Benzonase and Cyanase preferring GC-rich sequences and disfavoring AT-rich sequences.

Currently, formaldehyde-assisted isolation of regulatory elements (FAIRE) is the only reported method to identify regulatory elements in frozen tissue samples [23]; however, FAIRE is an ambiguous approach that relies on the density of proteins at regions of crosslinked chromatin, where less dense regions are identified by FAIRE [24]. As a consequence, FAIRE signals depend greatly on crosslink efficiencies of the tissue material, sonication efficiency and organic extraction steps. FAIRE shows some degree of overlap with DNase I accessible regions, however, certain regions are FAIRE specific and many regions are not identified by FAIRE [25]. Since the TACh procedure like the DNase I assay relies on nuclease accessibility of DNA within chromatin, TACh is a more reliable procedure that is less dependent on extrinsic variables.

For clinical samples, TACh provides several advantages compared to current nuclease based methods. 1) TACh uses frozen tissues, which eliminates the need for immediate processing of freshly acquired tissue and is, therefore, compatible with the acquisition of samples under routine clinical conditions. 2) The ability to store whole tissue, fragments or pulverized powders provides flexibility and the powder ensures easy generation of matched aliquots that can be used for additional genomic experiments. 3) The ability to interrupt the procedure at multiple steps makes the whole process more amenable to clinical situations and laboratories. 4) The quick processing and the use of whole cells are likely to minimize nuclear damage and the loss of dissociated chromatin components. 5) Because the chromatin structure is preserved, it is likely that the same pulverized tissue can serve as the starting material for ChIP-Seq interrogations of the same tissue.

Thus TACh will make it possible to mine the accessible genomes from banks of frozen specimens, including biopsies and resected material from diseased and healthy human tissue, and is likely to assist in understanding the pathophysiology of a number of disease states. In particular, since TACh efficiently identifies accessible CpG islands, the method may be used to characterize chromatin structure rearrangements during progression of cancer, which are often associated with abnormal DNA methylation at CpG island rich promoters, leading to deregulation of numerous genes [26]. Identification of genomic regions that specifically change accessibility during tumorigenesis may have significant prognostic value.

## Conclusion

The described TACh methodology is a robust method for highly sensitive and comprehensive detection of accessible chromatin in samples derived from frozen tissue. The robustness and quick processing time of the assay provides feasible analysis of multiple tissue biopsies and we propose that application of TACh on clinically derived tissue material will provide knowledge on changes in chromatin accessability during progression of diseases.

## Methods

### Cell culture

HL-60 (ATCC) suspension cells were cultured in RPMI + 10% FBS in CO2 at 37°C.

### Animals

Male C57BL/6 mice, 8-week old purchased from Harlam Sprague Dawley (Frederick, MD), were maintained according to the NIH guidelines with a 14-h light, 10-h dark cycle and free access to food and water. Mice were killed by decapitation or cervical dislocation and livers were immediately sectioned into 5-10 mm cubes and frozen in liquid nitrogen or processed for nuclei purification. All animal procedures were approved by the Animal Users and Care Committee, NICHD and NCI, NIH.

### TACh on whole cells

Cells were collected by centrifugation, washed twice in ice cold cellular wash buffer (CWB) (20 mM TrisHCl pH 7.5, 137 mM NaCl, 1 mM EDTA , 10 mM sodium butyrate, 10 mM sodium orthovanadate, 2 mM sodium fluoride, protease inhibitor cocktial (Roche)) and resuspended in (40 million cells/ml) hypertonic lysis buffer (HLB) (20 mM TrisHCl pH 7.5, 2 mM EDTA, 1 mM EGTA, 0.5% glycerol, 20 mM sodium butyrate, 2 mM sodium orthovanadate, 4 mM sodium fluoride, protease inhibitor cocktial (Roche)). Cells were distributed in 500ul aliquots in 1.5 ml tubes and following addition of 500ul of nuclease digestion (ND) buffer (40 mM TrisHCl pH 8.0, 6 mM MgCl2, 0.3% NP-40, 1% Glycerol) containing a 3-fold dilutions (from 0.125 units/ml to 6U units/ml) of Benzonase (Calbiochem/EMD) or Cyanase (RiboSolutions, Inc). This was mixed gently and incubated for 3 minutes at 37°C. Reactions were terminated by the addition of EDTA (10 mM final) and SDS (0.75% final). Proteinase K was added to a final concentration of 0.5 mg/ml and incubated overnight at 45°C. DNA was purified by Phenol/Chloroform/isoamyl (PCI) extraction, ethanol precipitated and processed for Southern blotting.

### Southern blotting

Purified nuclease digested DNA was digested with HindIII overnight and then PCI extracted and ethanol precipitated. Precipitated DNA was SacI digested, RNase treated, extracted by PCI and ethanol precipitated. Digested DNA was separated on an agarose gel and transferred to a nylon membrane and incubated with a biotinylated c-myc probe [14] overnight at 50°C. Membranes were washed, incubated with HRP-conjugated streptavidin solution (kit from KPL, Inc.) and developed using chemiluminescent reagents (ECL Plus from Amersham/GE HealthCare).

### TACh on frozen liver tissue

Livers were rapidly removed from mice and frozen immediately in liquid nitrogen. Frozen liver fragments were pulverized using a stainless steel pulverizer (Bessman), pre-chilled in liquid nitrogen. Seven hundred mg of frozen (the procedure may be done on a smaller amount of starting material), pulverized tissue was transferred to a pre-cooled 15 ml tube and suspended in 4 ml HLB (30 mM TrisHCl pH8.0, 2 mM EDTA, 2 mM EGTA, 20 mM sodium butyrate, 2 mM sodium orthovanadate, 4 mM sodium fluoride, protease inhibitor cocktail (Roche)). The suspension was mixed with a 3 cc syringe fitted with a 19 gauge needle and HLB was added to a final volume of 7 ml. The resulting suspension was passed through a 19 gauge needle 5 times, followed by a 22 gauge needle 5 times and finally a 23 gauge 10 times, using a 3 cc syringe. The suspension was distributed in 500ul aliquots in 1.5 ml tubes using a 23 gauge needle and incubated on ice for 5 minutes. 500ul of ND buffer (30 mM TrisHCl pH 8.0, 14 mM MgCl2 0.5% NP-40, 0.2% fatty acid free BSA) containing a 2-fold dilutions of Benzonase (Calbiochem/EMD) or Cyanase (RiboSolutions, Inc) was mixed gently with the 500ul tissue solution and incubated for 3 minutes at 37°C. Reactions were terminated by the addition of a final concentration of 50 mM EDTA and 0.1% SDS. 50ul RNaseA/RNaseT1 (Ambion) was added and the reaction was incubated over night at 40°C. SDS was brought to a final concentration of 0.75% and incubated for 2 hours at 45°C, after which Proteinase K was added (0.8ug/ml final) and incubated overnight at 45°C. DNA fragments of 100-500 bp from a chromatin digestion were purified over sucrose gradients [1] and precipitated in 0.1 volume NaAc and 0.7 volume isopropanol.

### DNase I digestion of chromatin from liver tissue

Livers was removed from mice and homogenized in 8 ml/g tissue of low sucrose buffer (250 mM sucrose, 15 mM Tris–HCl pH 8.0, 15 mM NaCl, 60 mM KCl, 1 mM EDTA, 0.5 mM EGTA, 1 mM spermidine, protease inhibitors (Roche)) using a Type B dounce. Crude nuclear pellets were washed once in low sucrose buffer and resuspended in 9 volumes of high sucrose buffer (2 M sucrose, 15 mM Tris–HCl pH 8.0, 15 mM NaCl, 60 mM KCl, 1 mM EDTA, 0.5 mM EGTA, 1 mM spermidine, protease inhibitor cocktail (Roche)). The nuclear suspension was aliquoted in 2.0 ml tubes and centrifuged at 16,000xg for 30 minutes at 4°C. Nuclear pellets were combined in buffer (15 mM Tris–HCl pH 8.0, 15 mM NaCl, 60 mM KCl, 1 mM EDTA, 0.5 mM EGTA, 1 mM Spermidine, protease inhibitor cocktail (Roche)) and DNase I digestions were performed with 20 million nuclei as previously described ([1]. DNA fragments of 100-500 bp from a chromatin digestion with 60U/ml DNase I (Sigma) were purified using sucrose gradients [1] and precipitated in 0.1 volume NaAc and 0.7 volume isopropanol.

### Sequencing and data analysis

DNA was sequenced using Illumina GII sequencer at the Advanced Technology Center, NCI (Rockville, Md). Sequenced DNA was aligned to the mouse genome (mm9) using Eland or Bowtie [27]. Hotspots were identified as previously described [1,4,28] using a Fdr of 0% and a tag density threshold at the mode. Hotspots were identified either from tag libraries generated from individual concentrations of Benzonase, Cyanase or DNase I or tag libraries pooled from different concentrations of enzymes or replicates of the same concentration. Bioinformatic analysis was performed using HOMER [29], Galaxy [30] and tools described in [4,31]. Sequence data is accessible at GEO: GSE39982.

### Previous published data used for analysis

RNA-seq [18]; SREBP-2 ChIP-seq peaks [22]; FOXA2 ChIP-seq peaks [32]; MNase-seq tag library (GEO: GSM717558) [16]; H3K4me1 and H3K4me3 ChIP-seq tag library (SRA008281) [15]; H3K27me2 ChIP-seq tag library (GEO: GSM751034). 

## Abbreviations

bp, Base pair; ChIP, Chromatin Immunoprecipitation; ChIP-seq, ChIP combined with massive parallel sequencing; DNase-seq, DNase I digestion of chromatin combined with massive parallel sequencing; FAIRE, Formaldehyde assisted identification of regulatory elements; FOXA2, Forkhead box A2; H3K4me1, Histone3 lysin 4 mono-methylation; H3K4me3, Histone3 lysine 4 tri-methylation; H3K27me3, Histone3 lysine 27 tri-methylation; Mnase, Micrococcal nuclease; RNA-seq, Massive parallel sequencing of RNA; SREBP, Sterol Regulatory Element-Binding Protein; TACh, Tissue accessible chromatin; TSS, Transcriptional start site.

## Competing interests

The authors declare that no competing interests exist.

## Authors’ contributions

LG carried out the DNase-seq in liver, participated in TACh-seq in liver, analyzed sequencing data and drafted the manuscript. RB developed the TACh method and performed TACh in cell lines and liver tissue, SJ participated in DNase-seq and TACh-seq in liver and helped to draft the manuscript. SB analyzed sequencing data. HJC helped develop the TACh method. YL and GA performed all the mouse work. CO and GLH participated in the study design and GLH helped to the draft of the manuscript. DL designed and coordinated the study and helped draft the manuscript. All authors read and approved the manuscript.

## Supplementary Material

Additional file 1 **Figure S1. **Table of aligned sequence tags. Figure S2: Correlation of sequence tag densities from chromatin digested with different concentration of Cyananse. Figure S3: Correlation of sequence tag densities from chromatin digested with Benzonase and Cyanase. Figure S4: Distribution of AT percentage surrounding DNase I, Benzonase and Cyanase hotspots. Click here for file
