# Supplementary material for: Rapid genome-scale mapping of chromatin accessibility in tissue
Source: Epigenetics Chromatin. 2012 Jun 26;5:10. doi: 10.1186/1756-8935-5-10 (PMC3464876; doi:10.1186/1756-8935-5-10)
Supplement: Additional file 1 — Figure S1. Table of aligned sequence tags. Figure S2: Correlation of sequence tag densities from chromatin digested with different concentration of Cyananse. Figure S3: Correlation of sequence tag densities from chromatin digested with Benzonase and Cyanase. Figure S4: Distribution of AT percentage surrounding DNase I, Benzonase and Cyanase hotspots. [file 1756-8935-5-10-S1.pdf]

Grontved_Supplementary_Fig1

Sample	Aligned tags (millions)	Aligned tags to mito genome (millions)
DNase I (rep1)	27.8	0.33
DNase I (rep 2)	27.0	0.44
Benzonase 0.25U/ml	21.7	3.52
Benzonase 1U/ml	22.3	4.21
Benzonase 4U/ml	23.4	3.98
Cyanase 0.25U/ml	17.1	3.83
Cyanase 1U/ml	20.7	4.91
Cyanase 4U/ml	23.9	6.43

Grontved_Supplementary_Fig2

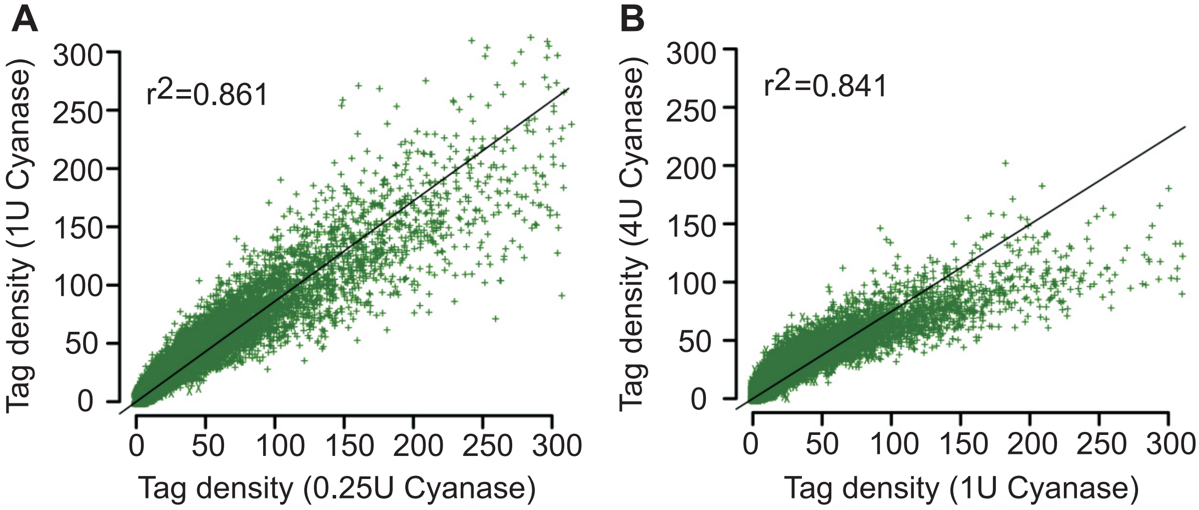

Grontved_Supplementary_Fig3

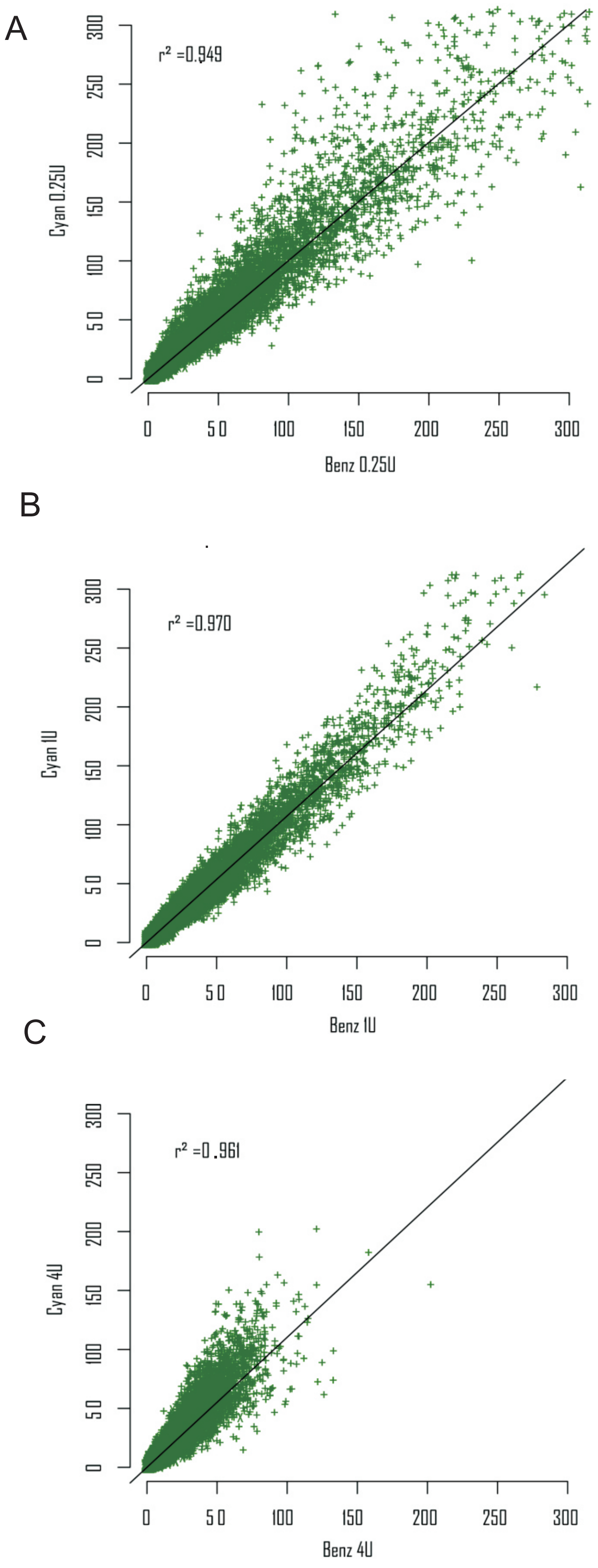

Grontved_Supplementary_Fig4

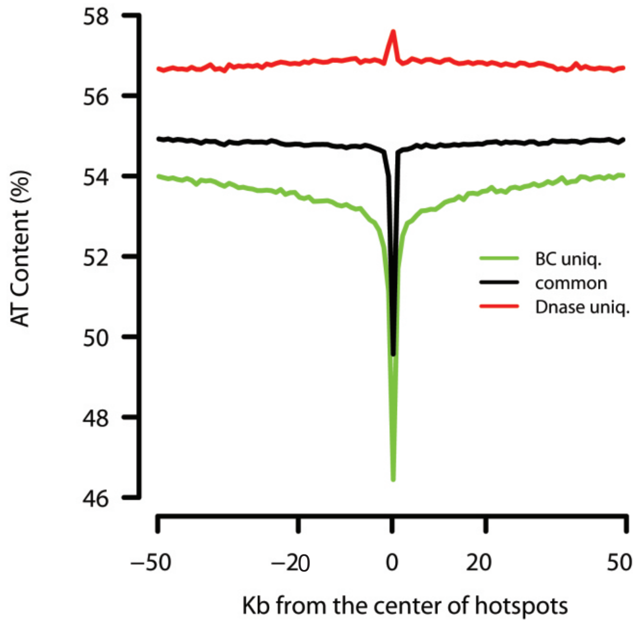

Supplementary figure legends_Grontved et al

Supplementary figure 1. Aligned tags from DNase I, Benzonase and Cyanse digestions of chromatin from nuclei and frozen mouse liver tissue. Tags were aligned to mm9 and to the mitochondrial (mito) genome.

Supplementary figure 2. Scatter plot of hotspot tag density from liver tissue digested with Cyanase. (A) 0.25U/ml vs. 1U/ml. (B) 1U/ml vs. 4U/ml.

Supplementary figure 3. Scatter plot of hotspot tag density from liver tissue digested with different concentrations of Benzonase and Cyanase. (A) 0.25U/ml Benzonase vs. Cyanase. (B) 1U/ml Benzonase vs. Cyanase. (C) 4U/ml Benzonase vs. Cyanase.

Supplementary figure 4. Distribution of AT percentage surrounding DNase I/Benzonase/Cyanase (common), DNase I unique and Benzonase/Cyanase unique hotspots.
